# A Case of Birt-Hogg-Dubé Syndrome and Multiple Intracranial Aneurysms

**DOI:** 10.7759/cureus.6884

**Published:** 2020-02-05

**Authors:** Nikhil K Murthy, Matthew B Potts, Babak Jahromi

**Affiliations:** 1 Department of Neurological Surgery, Northwestern University Feinberg School of Medicine, Chicago, USA

**Keywords:** cerebral aneurysm, birt-hogg-dube syndrome, flcn, folliculin, matrix metalloproteinase-9, mmp-9

## Abstract

Birt-Hogg-Dubé (BHD) syndrome is a rare autosomal dominant condition that is associated with fibrofolliculomas, pulmonary cysts, renal cysts, and renal cancer. There have been few reports in the literature of intracranial vascular pathology in patients with BHD syndrome, and intracranial vascular pathology is currently not a part of the diagnostic criteria. Given the rarity of this disease, there has not been enough evidence for a definitive link between BHD syndrome and intracranial vascular abnormalities. We present a case of a patient with BHD syndrome and multiple cerebral aneurysms.

## Introduction

Birt-Hogg-Dubé (BHD) syndrome is a rare autosomal dominant condition that can present with skin lesions, pulmonary cysts, spontaneous pneumothorax, and renal cancer. It is associated with a mutation in the FLCN gene, a tumor suppressor gene that codes for the protein folliculin. More than 600 families have been diagnosed with BHD syndrome, but there are few reports in the literature describing an association between BHD syndrome and intracranial vascular pathology [1]. So far, these cases include two patients with intracranial aneurysms, one with an arteriovenous malformation (AVM) and one with carotid aplasia [2]. There is a hypothesized link between the BHD gene mutation and vascular pathology, although this is yet to be proven definitively [3-5]. Here we describe a case of a patient with BHD syndrome and multiple intracranial aneurysms.

## Case presentation

**Figure 1:**
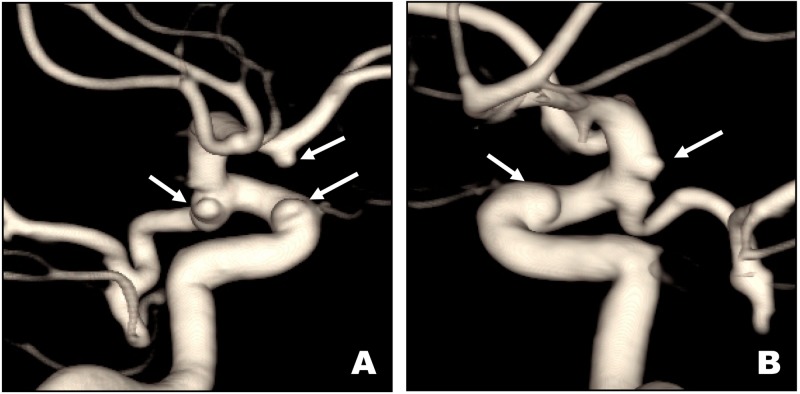
ICA Aneurysms Digital subtraction angiography three-dimensional reconstructions of right (A) and left (B) internal carotid artery (ICA) injections demonstrating small paraclinoid and posterior communicating arteries of the right ICA (A) and small paraclinoid and anterior choroidal artery aneurysms of the left ICA (B).

A 61-year-old female with BHD syndrome was referred to our clinic after workup of headaches and confusion revealed multiple cerebral aneurysms on magnetic resonance imaging (MRI). These findings were confirmed on follow-up digital subtraction angiography, which revealed a 2-mm broad-based right paraclinoid internal carotid artery (ICA) aneurysm, a 2-mm right posterior communicating artery aneurysm, a 2-mm left paraclinoid ICA aneurysm, and a 2-mm left anterior choroidal artery aneurysm (Figure 1). There was also an infundibular anterior communicating artery aneurysm on the right. She has no history of known aneurysm rupture or sudden onset of a severe headache, and her MRI showed no evidence of subarachnoid hemorrhage. 

The patient has a family history of BHD syndrome (aunt and two cousins), and her own clinical diagnosis was based on the presence of numerous fibrofolliculomas, as well as pulmonary, renal, and hepatic cysts. Of note, her aunt also reportedly had an unruptured cerebral aneurysm. The patient smoked a few cigarettes per day between the ages of 16 and 21 years, although she has not smoked since then. She remains asymptomatic of any respiratory symptoms related to BHD syndrome and is able to exercise daily. She remains stable with respect to her syndrome and has undergone serial non-invasive cerebrovasculature imaging for three years demonstrating no change in the size or morphology of her intracranial aneurysms.

## Discussion

The low frequency of cases of BHD syndrome makes it difficult to find a definitive association with intracranial vascular pathology. Previous case reports have reported a female who presented at age 50 years with subarachnoid hemorrhage from a ruptured intracranial aneurysm, a female who presented at age 25 years with an intraparenchymal hematoma from a ruptured AVM, and a female who was diagnosed at age 18 years with a cerebral aneurysm found on screening MRI done after genetic testing revealed an FLCN mutation [3]. Given the limited number of cases, there has been little research on the causative mechanism of intracranial vascular pathology that can specifically occur in BHD syndrome.

The FLCN mutation that is responsible for BHD syndrome affects folliculin transcript products and has been shown to have downstream expression in the brain [6,7]. One hypothesis for association between BHD syndrome and intracranial aneurysms is the association between the FLCN mutation and downstream effects on hypoxia-inducible factor and consequently aneurysm formation [4,5,8]. FLCN mutations have also been associated with abnormal matrix metalloprotease 9 (MMP-9) activity, which in turn has also been associated with cerebral aneurysms [9,10]. These associations between FLCN and cerebral aneurysms are suggestive of a potential mechanism of aneurysm formation in BHD syndrome, although there are no specific studies showing this mechanism in this specific patient population.

## Conclusions

The risk of aneurysm formation or rupture in this population is unknown given the low frequency of patients with BHD syndrome. There are proposed mechanisms for the association between BHD syndrome and cerebral aneurysms, although these have not been directly proven. Other known risk factors for cerebral aneurysms continue to include family history (including other hereditary syndromes), smoking history, and hypertension. We recommend that patients with BHD syndrome undergo a careful and thorough family history with respect to intracerebral vascular pathologies such as a history of aneurysms and AVMs, as well as any history of intracerebral or subarachnoid hemorrhage. However, more data regarding the incidence of cerebral aneurysms in BHD syndrome are needed, in order to determine the efficacy of screening with vascular imaging in this patient population.
